# A donor twin discordant with Peters anomaly in a twin–twin transfusion syndrome case: a case report

**DOI:** 10.1186/s12884-020-03269-0

**Published:** 2020-09-23

**Authors:** Yao-Lung Chang, An-Shine Chao, Ching-Yu Chou, Shuenn-Dyh Chang, Ming-Chou Chiang, Yung-Sung Lee

**Affiliations:** 1 Department of Obstetrics and Gynecology, Chang Gung Memorial Hospital, Chang Gung University College of Medicine, Tao-Yuan, Taiwan; 2 Department of Obstetrics and Gynecology, Cathay General Hospital, Hsinchu, Taiwan; 3 Department of pediatrics, Chang Gung Memorial Hospital, Chang Gung University College of Medicine, Tao-Yuan, Taiwan; 4 Department of Ophthalmology, Chang Gung Memorial Hospital, Linkou, Taiwan

**Keywords:** Peters anomaly, Monochorionic twin, Case report

## Abstract

**Background:**

Peters anomaly is a rare form of anterior segment ocular dysgenesis, the antenatal image of Peters anomaly had not been reported. We herein showcased a discordant finding of Peters anomaly in a monozygotic twin complicated with twin-twin transfusion syndrome (TTTS) and exhibited its antenatal sonographic images,

**Case presentation:**

**A** 38-year-old gravida 2 para 1 pregnant woman visited our clinic at the gestational age of 18 weeks where TTTS stage III was diagnosed and the following laser therapy was done successfully. Ten days after the surgery, the follow-up ultrasound detected the opacity of both fetal eyeballs in the donor twin and thus congenital cataract was suspected initially. Then magnetic resonance imaging (MRI) examination was arranged at the gestational age of 23 weeks, and no central nervous system or other anomaly was found. At the 29 weeks of gestation, the opacity of both fetal eyeballs of the donor twin did not clear. The pregnancy resulted in cesarean section at the gestational age of 37 weeks indicated by malpresentation where two male live births were born. Examination under anesthesia was arranged for donor twin after delivery and Peters anomaly was diagnosed based on central corneal opacity with iridocorneal and corneolenticular adhesions.

**Conclusions:**

The prenatal image of Peters anomaly may present as the opacity of the fetal eyeballs similar to congenital cataract. Some cases of the Peters anomaly had been reported with a genetic abnormality, but since our case presented discordant presentation in monozygotic twin pregnancy where both twins are supposed to share the same genetic make-up, therefore other factors that are epigenetic may be held accountable. Nevertheless, a genetic origin of the anomaly in our case cannot be excluded.

## Background

Peters anomaly (PA) is a rare form of anterior segment dysgenesis characterized by corneal opacity with or without iridocorneal and/or corneolenticular adhesions [1]. It has been subdivided into type I and type II and Peters plus syndrome [2]. Peters plus syndrome is associated with cleft lip/palate, short stature, abnormal external ears and mental retardation [2]. The systemic anomalies of Peters plus syndrome can be detected by prenatal image study like sonography, [3] but PA type I and type II, owing to being characterized by localized ocular abnormalities, were never reported with prenatal image diagnosis. Some PA cases had been studied to reveal genetic defects [1], but a majority of them lacked a genetic diagnosis [4]. We reported a case of monohorionic twin complicated with twin-twin transfusion syndrome (TTTS). The prenatal sonography 10 days after fetoscope-guided laser therapy showed opacity of fetal eyeballs in the donor twin and after delivery, the twin was diagnosed with discordant PA.

## Case report

A 38 -year-old gravida 2 para 1pregnant woman was referred to our prenatal clinic for a survey at 18 weeks of gestation due to twin pregnancy with the discordant amniotic fluid amount. Sonographic examination revealed polyhydramnios with a maximum vertical pocket (MVP) of 12 cm in the recipient fetus’s sac and another stuck twin fetus showing an absence of end-diastolic velocity of umbilical artery (UA) Doppler flow and thus a stage III TTTS was diagnosed. After consultation with the family about treatment options including amnioreduction and laser therapy, the patient chose to receive fetoscope-guided laser therapy. Under local anesthesia and following procedures as previously described [5], ten intertwin anastomoses (six donor artery to recipient vein anastomoses and four recipient vein to donor artery anastomoses) were coagulated with 10 ~ 15 W Diode laser power with Solomen technique. The postoperative course was uncomplicated and the UA Doppler returned to normal in donor twin after the surgery.

**Fig. 1:**
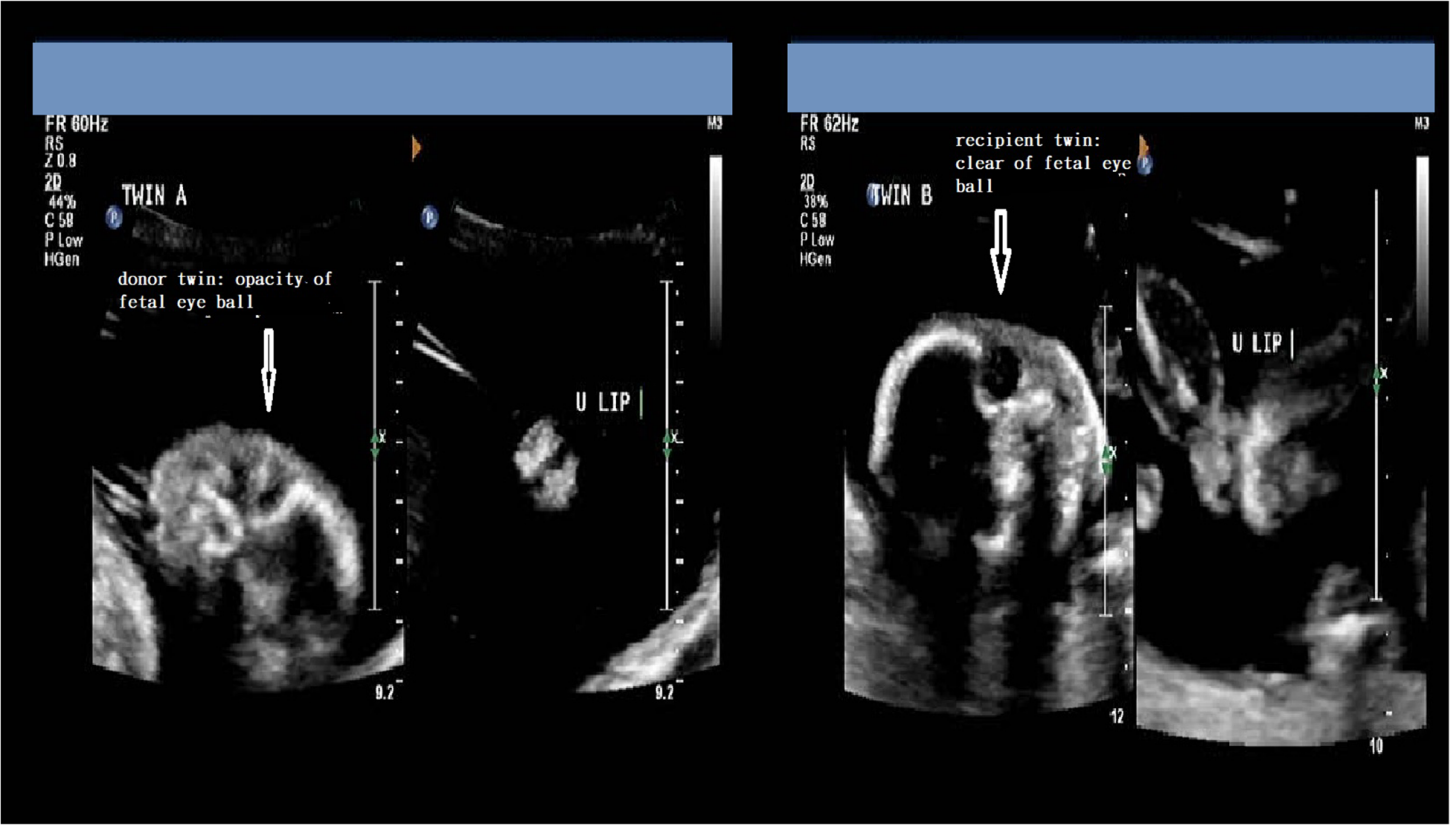
Opacity of donor twin (twin **a**) eye ball and clear eye ball of recipient twin (twin **b**) found ten days after laser therapy

**Fig. 2:**
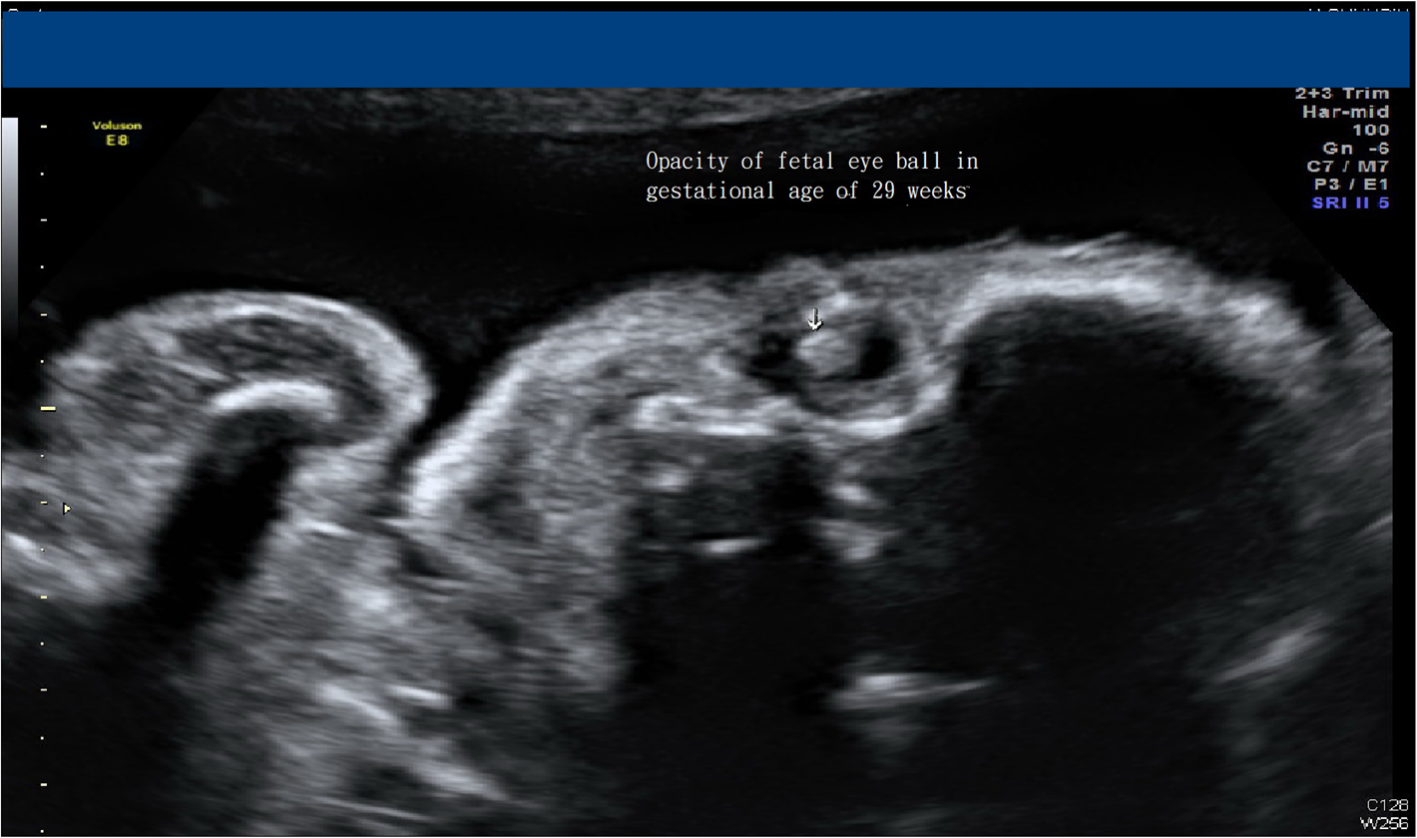
Opacity of donor twin (twin **a**) eye ball still be found at gestational age of 29 weeks

Ten days after the laser surgery, high-level ultrasound was done at the previous hospital that referred the case. Both fetuses had their amniotic fluid returning to normal, but incidentally they found both eyeballs of the donor twin were opaque, while the recipient’s were clear. (Fig. 1) Therefore, discordant congenital cataract was suspected in the donor twin. At the gestational age of 23 weeks, fetal magnetic resonance imaging (MRI) was arranged to evaluate the central nervous system for both fetuses and to rule out if the donor twin had other associated anomalies; no additional information was gathered about the donor twin. At the gestational age of 27 weeks, sonography still revealed opacity over donor twin eyeballs. (Fig. 2) The pregnancy was completed at gestational age 37 weeks by cesarean delivery indicated by malpresentation, resulting in the birth of two male babies, both babies weighing 2460 g each. Ten days after delivery by examination under anesthesia, the slit lamp biomicroscopy detected an annular opacity in the central cornea over the bilateral eyes of the donor twin. Ultrasound biomicroscopy also revealed extensively iridocorneal and corneolenticular adhesions, hence establishing the diagnosis of PA. Since patients with PA have a higher likelihood of glaucoma or amblyopia during infancy or toddler period, this PA baby was regularly followed up at our ophthalmology clinic. Trabeculotomy or angle surgery will be performed if glaucoma occurs; alternative treatments include medications and cyclodestructive procedures. Corneal transplantation is also a feasible option for this baby, depending on the extent of amblyopia, to improve his vision.

## Discussion and conclusion

We report a case of discordant PA in a monochorionic twin pregnancy complicated with TTTS, where the prenatal ultrasound images discovered opacity of donor eyeballs.

PA occurs between the 6th and 9th week of gestational age due to faulty separation of the lens from the surface ectoderm or aberrant reattachment of the lens/iris to the cornea during the development of the anterior chamber [6]. PA is characterized by the presence of a central corneal opacity at birth. It has been subdivided into type I and type II [2]. Type I PA is characterized by iridocorneal adhesions, and type II PA is recognized by central corneal opacity with cataracts or corneolenticular adhesions Our case is a severe form of PA with both iridocorneal and corneolenticular adhesions. The systemic involvement with PA can vary. The term Peters plus syndrome refers to PA associated with cleft lip/palate, short stature, abnormal external ears and mental retardation [2]. The systemic anomalies of Peters plus syndrome can be detected by prenatal image study like sonography, [3], but PA type I and type II, owing to being characterized by localized ocular abnormalities, were never reported with prenatal image diagnosis. In our case, the opacity of fetal eyeballs was found under sonographic examinations at the gestational age of 20 weeks, and the following MRI investigation revealed no other anomalies, thereby rendering Peters plus syndrome an unlikely diagnosis. Thus, the opacity of fetal eyeballs as detected by prenatal ultrasound not only served as a clue to congenital cataract, but to PA as well, in spite of its rarity. However based on ultrasound images, it is difficult to distinguish between the opacity of cornea and the cloudiness of the lens. Even fetal MRI has no role to play in this regard. Consequently, in our case, it was rather difficult to make a definite diagnosis of PA prenatally.

In literature, there was ever one discordant donor PA reported in a TTTS case, [7] where the PA was diagnosed after delivery without a prenatal image. TTTS is a complication of monochorionic twin pregnancies, so both fetuses share nearly the same genetic make–up; the concordance rate for any birth defect is higher in monozygotic twin pairs compared with dizygotic twin pairs [8]. Though a portion of PA had been reported associated with a genetic abnormality, [1] a majority of cases lack a genetic diagnosis [4]. Judging from our case and previous reports on the discordant PA in monochorionic twins, [7] we suspected epigenetic factors may play a part in the occurrence of PA. In our case and previous cases reported [7], the affected PA fetuses in TTTS were both donor twins; donor twins usually have relative hypotension and hypo-perfusion than recipient co-twin due to unequal placenta sharing and imbalanced inter-twin perfusion. So hypo-perfusion from the placenta could pose as one epigenetic cause of PA. Other epigenetic factors like different activation of maternal and paternal genes, [9] or genetic defects with de-novo mutations in one twin after splitting [10] should also be considered. In the absence of a molecular study, a future genetic workup may be proposed to the families to evaluate if any genetic causes of PA were involved in this case.

The strength of this case report is presenting prenatal ultrasonography images of isolated PA (Type I or II PA), that have not been reported before, and also revealing a discordant malformation in one monozygotic twin leads us to speculate that the origin of PA in this case may be due factors that have an epigenetic root. The weakness of this report is that without molecular study, whether there is a genetic cause for PA in this case couldn’t be ruled out.

In conclusion, our case has demonstrated PA should be placed among the differential diagnoses when prenatal ultrasound image revealed opacity of fetal eyeballs. Discordant PA in monochorionic twins highlights the possibility of an epigenetic cause of PA in these rare cases.

## Data Availability

The datasets obtained and/or analyzed during the current study are available. from the corresponding author on reasonable request.

## Abbreviations

PA: Peters anomaly
TTTS: Twin-twin transfusion syndrome
MVP: Maximum vertical pocket
MC: Monochorionic
UA: Umbilical artery
MRI: Magnetic resonance imaging
